# Directed Partial Correlation: Inferring Large-Scale Gene Regulatory Network through Induced Topology Disruptions

**DOI:** 10.1371/journal.pone.0016835

**Published:** 2011-04-06

**Authors:** Yinyin Yuan, Chang-Tsun Li, Oliver Windram

**Affiliations:** 1 Cancer Research UK, Cambridge Research Institute, Cambridge, United Kingdom; 2 Department of Oncology, University of Cambridge, Cambridge, United Kingdom; 3 Department of Computer Science, University of Warwick, Coventry, United Kingdom; 4 Warwick Horticulture Research Institute, University of Warwick, Wellesbourne, United Kingdom; Fondazione Telethon, Italy

## Abstract

Inferring regulatory relationships among many genes based on their temporal variation in transcript abundance has been a popular research topic. Due to the nature of microarray experiments, classical tools for time series analysis lose power since the number of variables far exceeds the number of the samples. In this paper, we describe some of the existing multivariate inference techniques that are applicable to hundreds of variables and show the potential challenges for small-sample, large-scale data. We propose a directed partial correlation (DPC) method as an efficient and effective solution to regulatory network inference using these data. Specifically for genomic data, the proposed method is designed to deal with large-scale datasets. It combines the efficiency of partial correlation for setting up network topology by testing conditional independence, and the concept of Granger causality to assess topology change with induced interruptions. The idea is that when a transcription factor is induced artificially within a gene network, the disruption of the network by the induction signifies a genes role in transcriptional regulation. The benchmarking results using GeneNetWeaver, the simulator for the DREAM challenges, provide strong evidence of the outstanding performance of the proposed DPC method. When applied to real biological data, the inferred starch metabolism network in *Arabidopsis* reveals many biologically meaningful network modules worthy of further investigation. These results collectively suggest DPC is a versatile tool for genomics research. The R package DPC is available for download (http://code.google.com/p/dpcnet/).

## Introduction

In recent years various multivariate analysis techniques have been developed for inferring causal relations among time series. Although many of them have previously proved their power on analysing economic and neurophysiological data, the unique nature of gene expression time series, typically large-scale and small-sample, poses a challenge to these techniques. On the other hand, gene expression dynamics are important, since they directly reveal the active components within the cell over time, indicating gene regulatory relationships at the transcriptional level. Therefore, a lot of time and effort has been spent on developing tools that suit the need for expression time series analysis.

**Figure 1:**
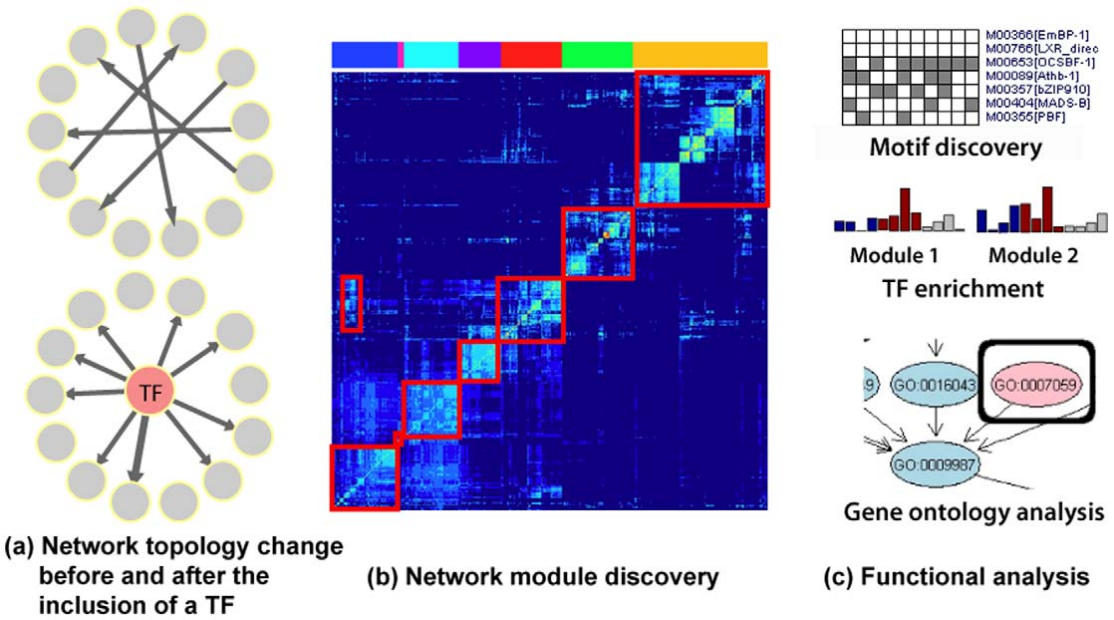
DPC for large-scale transcriptional regulatory network inference. (a) DPC detects network topology changes with the addition of a gene, the inclusion of a transcription factor should lead to dramatic changes of the connectivity of its downstream targets, (b) module discovery in the large-scale DPC network by biclustering the network adjacency matrix, (c) functional analysis of the network modules reveals putative transcription factors active under the biological condition.

We define a causal relation as a target at the current time having directed dependence on a regulator at the past time, when conditioned on the rest of the regulators (Figure 1(a)). Inferring causal relations between variables, when applied on gene expression data, is equivalent to inferring transcriptional regulatory relationships. Collectively, the complete set of regulatory relationships among genes leads to the reconstruction of gene regulatory networks. The resulting networks or network modules (Figure 1(b)), if evaluated together with biological knowledge, should provide new insights into the dynamics and functioning of the regulatory system (Figure 1(c)).

For example, a directed network inference approach, termed the shrinkage vector autoregressive method (SVAR), was proposed by Rhein *et al.* [1]. The class of shrinkage methods, which effectively shrink the effects from some predictors to zero, can both improve performance and reduce computational costs in many instances. In particular, SVAR is designed specifically for gene expression data to circumvent the small sample problem. Also, dynamic Bayesian networks (DBNs) [2], [3], a class of commonly used graphical models, have also been applied in this research area. Another recent advance in this area was the introduction of the concept of Granger causality [4] which is well known in economics for causal inference on time series data [5], [6]. For example, Zou *et al*. [5] compare DBNs and a method based on Granger causality and conclude that while the method based on Granger causality performs better with sufficiently large datasets (thousands of samples), DBNs are more likely to perform well on small-sample datasets (as is often the case in microarray experiments).

In this paper, we describe some of the most commonly used multivariate inference techniques for large-scale gene regulatory network reconstruction. We demonstrate that the proposed directed partial correlation (DPC) algorithm is an efficient and effective solution to causal/regulatory network inferences on small-sample, large-scale gene expression data. The comprehensive analysis of the experimental results not only reveals good accuracy of the proposed DPC method in large-scale prediction, but also gives much insight into all methods under evaluation.

**Figure 2:**
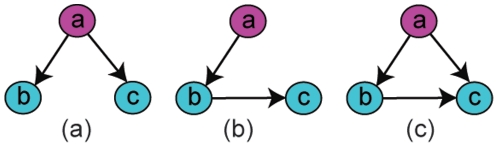
Possible inference results of the causal relations among three variables. (a) True/direct interactions, (b) indirect interaction inference, (c) bivariate inference.

In essence, partial correlation, which is able to test conditional independence on multivariate Gaussian data, is used as the mathematical foundation for establishing direct interactions among genes. For example, variable b is highly correlated with c because of the causal effects from a (Figure 2(a)). Pearson correlation may give rise to many false positives as in Figure 2(c), and Figure 2(b) may be probable for methods that do not account for conditional independence. However, partial correlation tests the correlation between two variables after the linear effects from the rest of the data are removed, hence no relationship exists between b and c after the effect from a is removed. (Note that partial correlation only infers undirected relationships, unlike what are shown in Figure 2.) Conditional independence, although by itself is insufficient to denote a causal link, can be a powerful tool for removing indirect relationships. Therefore, when inferring the relationship between two gene expression profiles, the other expression profiles can be taken into account to discriminate between direct (Figure 2(a)) and indirect (Figure 2(b) and (c)) interactions.

Although a shrinkage estimate of partial correlation [7] is computationally fast and well suited for small sample data analysis [8], the inferred interactions are undirected. In an undirected network, the role that a gene plays in different regulatory activities is unknown. Therefore, based on partial correlation, we propose a directed approach specifically targeted at small-sample gene expression data. It is then compared with some of the existing methods, DBNs, SVAR, and GC-VAR, to demonstrate its effectiveness.

An immediate difficulty in accessing a network inference method lies in the fact that current biological knowledge is far from sufficient to provide a clear picture. A reasonable validation process involves the use of real biological datasets, in addition to synthetic datasets which provide both ground truth and unlimited sample length. Under a broad set of assumptions, if datasets of various sample sizes and number of variables can be produced, an inference method then can be tested extensively, especially against its sensitivity to dimensionality. We adopt this validation process, but specifically note that, since most of the methods are probabilistic, selecting cutoffs to represent one resulting network may introduce false positives. Hence it is desirable to compare different methods with their direct output – the network probability matrix in which a coefficient denotes the probability of interaction between two genes.

The rest of this paper is organised as follows. In the second section, we present the technical details of the three existing algorithms, together with the proposed algorithm for directed regulatory network inference. Then benchmarking using datasets of various sizes generated by GeneNetWeaver [9] is presented. GeneNetWeaver provides simulations for DREAM (The Dialogue for Reverse Engineering Assessments and Methods) [10] *in silico* challenges. DREAM is a community effort to assess reverse engineering algorithms. Benchmarking using GeneNetWeaver datasets should provide strong evidence of the power of network inference algorithm in a controlled environment. Specifically, we discuss the statistical properties of transcriptional networks and their impacts on the performance of an algorithm in the comparative evaluation. In addition, we discuss model assumptions for different inference methods. The question is, to what extent the model assumptions influence the confidence of the inference outcome.

The experiments are designed to give a thorough evaluation of the proposed algorithm and to compare the four algorithms in a coherent manner. The reported results on simulated data indicate superior performance of the proposed algorithm both in terms of accuracy and efficiency. For the biological dataset, detailed analysis of the results suggests that DPC uncovers more biologically relevant regulatory relationships than the competing method SVAR.

## Methods

In this section, we hope to shed some light on the nature of different inference techniques, their advantages and inherent problems. First the autoregressive models are presented, they form the theoretical ground for most of the existing methods in comparison. Then we describe the technical details for the three representative existing methods, with notes on their capabilities in gene expression analysis. Next, the proposed DPC method is formulated. These technical details provide us with a strong foundation for later discussions of experimental results.

### Existing multivariate time series inference methods

#### Vector autoregressive models (VAR)

Suppose 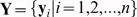 is a multivariate stationary time series consisting of 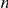 variables and 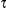 time points. A 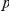 -order vector autoregressive VAR(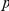) model specifies that the value of the 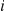 th variable at a time point 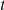, 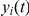, is a linear combination of a constant/mean value, the past of the multivariate time series, and a noise component
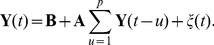
(1)



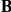
 is a constant matrix of length 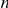. 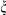 consists of vectors of residuals 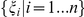, each assumed to be zero mean noise with variance 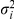. 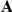 is the 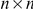 coefficient matrix representing the dynamic structure. When 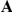 is a constant matrix, this model assumes homogeneity across time. A special case of the 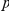 -order VAR process, the first-order autoregressive model (VAR(1)), is often considered when analysing short microarray time series [1], [3]

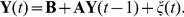
(2)


#### Granger causality inference method based on VAR model (GC-VAR)

Time series 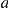 is said to Granger cause time series 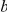 if the forecast of 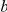 has incremental predictive power with the knowledge of the past state of 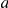 [4]. For the VAR models, a widely accepted measure of the predictive power of 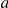 on 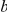 is the variance of the residuals as a result of model fitting [4], [11]. Informally, the method measures the influence of one time series on another by checking if the prediction of the response can be improved by incorporating the knowledge of the past of a predictor. One of the first attempts for gene expression data analysis is a bivariate model that uses Granger causality to infer relationships between pairs of variables without taking into account other variables [12]. For comparative purposes, we implemented a multivariate model, since the bivariate model could lead to false positive edges such as the ones in Figure 2(c), compared with the true network (Figure 2(a)).

In the multivariate case. Let 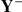 symbolise the past state of 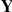, 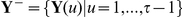, and let 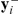 symbolise the past of variable 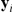. Based on Granger causality, the prediction power of one variable 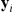 on the other variable 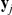, 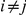, can be measured by
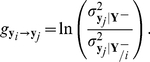
(3)


Symbol “
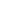
” denotes operation “condition on” and symbol “
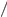
” denotes “without”. 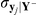 is the variance of the residual 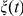 in the VAR(1) model for 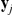 conditioned on the past of all variables 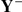. It is compared to 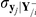 which is conditioned on the past of all variables but 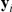, 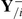. This method based on Granger Causality and the VAR model directly measures the prediction power of 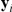 for 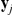, as a result of the reduction of prediction errors by incorporating 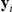 into the VAR(1) model for 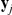. In other words, if introducing 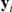 significantly reduces the variance of the prediction error of 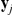, then a variable 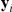 Granger causes the variable 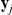. Since it requires fitting the autoregressive model with all variables and their past states, GC-VAR can only be applied to data satisfying: 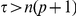, indicating its limited potential in gene expression analysis.

#### Shrinkage VAR method (SVAR)

Although the VAR model has been widely used in economics and neuroscience, it has its own limitations when small samples are encountered. An effective shrinkage estimation procedure was developed for learning the VAR models from small sample data [1]. The idea is that a shrinkage estimate can replace the covariance matrix for the joint matrix of both the present state and the past state(s), which then leads to the computation for regression coefficients. The basic procedure consists of first computing the shrinkage estimates of covariance matrices to obtain regression coefficients. Then instead of using the regression coefficients directly, the corresponding partial correlation coefficients are statistically tested. Significant coefficients are then selected using False Discovery Rate (FDR) [13] to be included in the reconstructed network.

The covariance matrix would otherwise be ill-conditioned, given the large number of variables (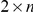) and short time series 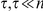. Let 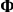 denote the joint matrix of the multivariate 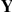 's present state (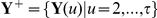) and past state with a time lag of 1 time point (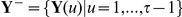), 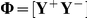. Assuming that the data has zero mean, an unbiased estimate of the covariance matrix for 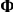 is
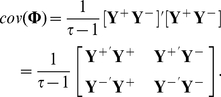
(4)


Note that this matrix contains the sub-matrices 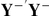 and 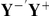. Meanwhile, the ordinary least squares (OLS) estimation [14] for the regression coefficient 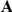 in the VAR(1) model (Eq. (2)) is:
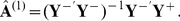
(5)


Therefore, the shrinkage estimation of 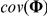 will lead to the estimated coefficient matrix 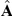. Then the partial correlation coefficients 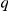 can be computed from 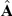 and the FDR is used to select significant coefficients. With large numbers of variables, this method gave good results in the comparative simulation study using simulated autoregressive data in the original paper [1].

#### Dynamic Bayesian Networks (DBNs)

DBNs are graphical models trained to maximise the joint probability of a set of observed data and their conditional dependencies. DBNs have been routinely applied to data, mainly long time series, to provide information about system dynamics. However, a major concern about DBNs is their inefficiency in large-scale prediction, i.e., with the presence of many variables.

DBNs implementations are usually designed for data with hundreds or thousands of samples. High costs of microarray experiments prohibit most of the techniques from exploring small sample gene expression data. In this paper, we use the implementation of the R package G1DBN [3], which is based on a trivariate AR(1) model:
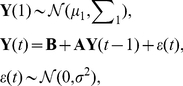
(6)with predefined 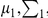 and 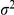. This method measures the conditional dependence between two variables 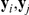 by testing the null hypothesis 
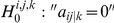
 on every third variable 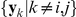. Then, a score is assigned to the potential edge 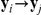 corresponding to the maximum 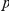 -values from the tests 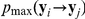. This means the algorithm has a computational complexity of 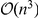. The computation of this method may be too heavy for data with more than a hundred variables.

### Proposed directed partial correlation inference method (DPC)

The shrinkage estimate for partial correlation in [7] was formulated specifically for the inference from small sample gene expression data. Although partial correlation is undoubtedly fast in computation and suitable for small sample problem, it can only infer undirected networks. Another problem is that variable time lag cannot be taken into account as in a VAR(1) model. We introduce the notion of directed partial correlation (DPC) for fast inference of directed gene networks. The idea is similar to the idea behind Granger causality – a variable 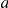 has causal influence on another variable 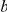, if the removal/addition of 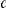 has a large impact on the prediction of 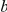. While GC-VAR measures this impact by comparing the residuals before and after adding 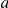 to the prediction of 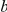, DPC measures it by examining the correlation coefficients.

#### Zero-order directed partial correlation DPC(0)

**Figure 3:**
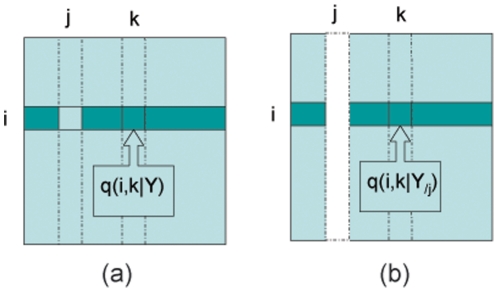
Partial correlation matrices before and after deleting 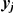. To predict 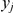
's influence on 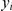, two groups of partial correlation coefficients from two matrices (coloured dark green) are tested. (a) Coefficients in 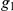, (b) Coefficients in 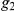.

Directed partial correlation aims to investigate the effect of including a variable in the prediction of another gene, i.e. the change of dependencies among other genes. Let 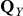 of size 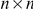 denote the partial correlation matrix for 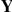. Each element 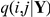 in 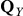 is the partial correlation between 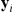 and 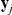 given 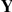, 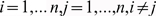, i.e., the correlation between 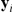 and 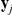 after the linear effects of the rest of the variables are removed. This can be formulated as 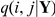. The removal of linear effects from others means that the resulting partial correlation indicates the direct relationship between two variables. Figure 3(a) shows 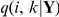, 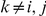, which denotes the partial correlation between 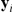 and 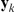 when effects from all others, including 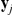, are removed.

However, the conditional dependence indicated by 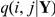 is undirected. To investigate the influence 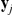 has on 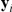, we propose the following. If we delete the variable 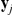 from 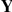, the partial correlation between 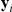 and another variable 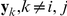 is denoted as 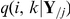 in the matrix 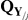. As shown in Figure 3(b), in the prediction of the relationship between 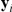 and any other variable 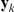, 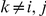, 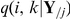 no longer remove the effect from 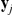, which means 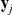 no longer take part in the prediction of 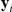. Consequently, there are two groups of statistics related to the prediction of 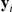, each corresponding to coefficients before and after the removal of 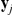. To be more specific, the first group is the 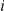 th row in 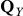 without the 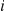 th and 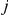 th element, 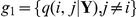, shown in dark green in Figure 3(a). The second group corresponding to the dark green elements in Figure 3(b) is the 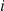 th row in 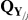 without the 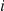 th element, 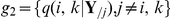. Both groups have the length of 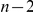. The effect 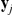 has on the prediction of 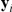 is defined as:
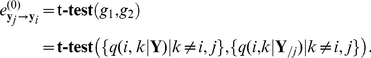
(7)


We use a paired t-test on the two groups to see if there is an effect on the prediction of other variables with the removal of variable 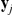. The null hypothesis is that there is no significant difference between the two groups, before and after the removal.

In summary, we take advantage of the fact that in computing partial correlation between two variables, all effects from other variables are removed. In other words, 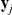 takes part in the predictions of 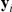 with all other variables 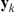. We measure 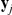 's influence on 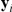 by comparing partial correlation coefficients related to 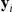 before and after the deletion of 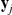, since 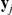 does not take part in the prediction of 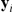 after the deletion.

#### 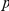 -order directed partial correlation DPC(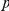)

A key feature of the proposed DPC method is that it can be easily extended to include time lags. In the following discussion, we focus on first-order DPC (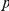  = 1) for the sake of simplicity, although the DPC algorithm can be generalized to any reasonable order 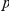. Note that 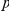 needs to be carefully chosen according to the microarray experimental design in order to capture the regulatory events.

Let 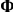 be the joint matrix of the present state and the past state of data, i.e., 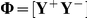. To compute the correlation matrix for 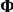, we note that the covariance matrix of 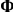 is ill-conditioned for small sample data and therefore not suitable. We use the shrinkage estimate method in Eq. (4) to compute the partial correlation matrix 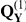 for 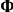


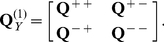
(8)


Hence each element in the sub-matrix 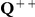, 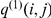 with 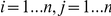, stands for the partial correlation between 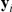 and 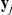, when the effects of the present states of other variables and the past states of all variables are removed. If a variable 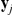 is deleted from the joint matrix 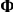, the corresponding partial correlation matrix 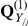 has an equivalent meaning as described in the zero-order model, i.e. the effect of 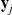 is not taken into account in the prediction of the other variables. The first-order directed partial correlation from 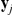 to 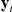 can be formulated as
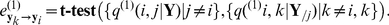
(9)


**Figure 4:**
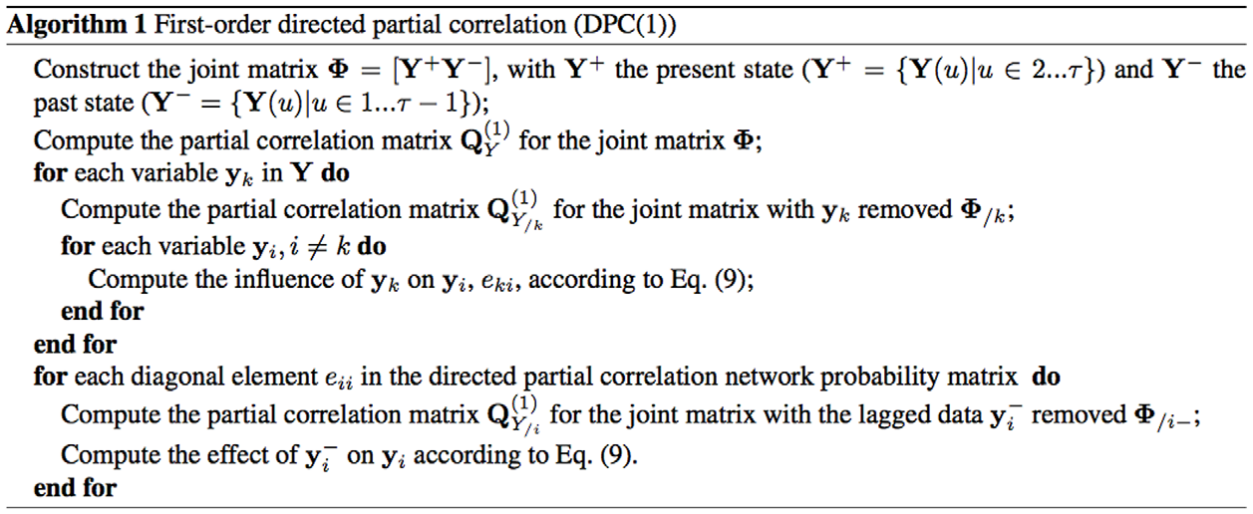
The algorithmic pipeline for first-order DPC. By avoiding linear model fitting it is thus more efficient and less constrained by the sample size.

Note that although the partial correlation matrix is of size 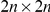, only the sub-matrix 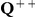 is used for computing 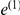. The probability of the directed interaction is indicated by the resultant 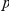 -values. Using FDR, adjusted 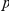 -values are selected in accordance with confidence levels, for example, 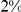 of FDR means accepting all tests with adjusted 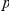 -values 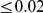 as significant. The algorithmic pipeline is described as in Figure.4.

Conceptually, DPC tests the effect of one variable on the predictions of another, whist taking into account all the rest of the variables at the same time. Hence it is able to monitor the dynamic process within reasonable computation time. It avoids linear model fitting and thus is more efficient and less constrained by the sample size. Note that a major difference between DPC and other methods is, while others inspect the regression coefficients of full linear models, DPC takes advantage of the concept of Granger causality, based on a computationally fast method.

## Results

### Experiments on synthetic datasets

Since the ground truth is unknown for real expression data, comparisons of performance are first conducted on synthetic data and then on biological data.

Previously, SVAR and DBNs were experimentally proved to be useful using simulated data from autoregressive models [1], [3]. These methods are based on the autoregressive model and their performance on other types of data is still not clear. When the data satisfies the model assumption, we can expect the corresponding technique to perform well. Therefore, an important question pertains to which assumption best describes gene expression data. In this section, we aimed to investigate the following question: how well the inference methods meet the requirements of microarray data?

The synthetic data generator GeneNetWeaver uses topologies generated based on real biological networks, therefore allowing good approximation of the statistical properties of real biological networks. It can sample from these transcriptional regulatory networks, and produce corresponding microarray datasets parameterized by the network topology, size of the network/number of genes, and type of experimental noise etc.

#### Network topology

Network topologies are generated by selecting sub-networks from a previously described *Ecoli* network. Neighbouring genes are selected randomly among top 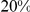 genes based on connectivity. This is to introduce stochasticity into gene selection. In this way, the resulting sub-network preserves features of scale-free networks such as modularity but it also allows the possibility of including small hubs and their targets. Consider, that one may want to model how the hub genes interact with their targets, but not all of the targets can be selected during the variable selection process. Therefore, sub-network generation by randomly selecting genes among the top 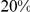 may represent a realistic situation in gene network analysis.

#### Kinetic model

After the topologies of the synthetic networks are sub-sampled from the *E. coli* transcriptional network, kinetic equations are selected for each gene and its regulators without removing the autoregulatory relations. Different types of perturbations are applied to the networks, including multifactorial, dual knockout, knockout, and knockdown. We choose to model the gene network with ordinary differential equations. With this system the perturbations are applied at 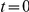 and the statistical properties of the network do not depend on time.

#### Experimental design

In each run, we apply a method in comparison to a single gene expression time series dataset, e.g., a dataset with multifactorial perturbation, 500 genes, and 21 time points. In the DREAM challenge a team uses datasets of all four types of perturbations and multiple simulations to collectively infer a network. The DPC algorithm however, was formulated to deal with large numbers of genes and few time points. We found this situation underrepresented in the DREAM challenge datasets. For this reason we chose to use the DREAM challenge data simulator (GeneNetWeaver) to provide a more appropriate dataset for the assessment of this method. The simulations in this paper represent even more difficult problems for the inference methods. Specifically, for knockout and knockdown experiments, a simulation may see the change of expression profiles of very few genes while others remain constant. For this to be a standalone test, we then select the datasets based on the variance in the dataset and only use datasets with high variations.

#### Parametrizing the simulations

We simulate networks of size 50, 100, 200, and 500 genes with four types of perturbations. The times series are all simulated from time point 1 to 100, but measured with 21 time points or 100 time points to form two datasets with different time series lengths. Experimental noise is modeled by simulating noise in microarrays, which is a mix of normal and log normal noise. Then the data is normalized after the experimental noise is added. With 4 network sizes, 4 types of perturbations, 2 time series lengths, and 5 simulations for each setup, there are altogether 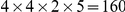 datasets.

#### Assessment metric

Four multivariate time series inference algorithms as described above are evaluated in this experiment. Their ways of inferring the final network vary and each requires fine tuning for the parameters, which could be subjective for large-scale experiments (altogether 142 synthetic datasets are used). To eliminate any subjective elements and enable a fair comparison, we decided to compare directly on their preliminary output, the network probability matrices. For clarity, the related symbols for each probability matrix in the algorithms' technical details are listed in Table 1.

**Table 1:** Average consumed time of the four multivariate time series inference algorithms on the 100 time point datasets.

Method	DPC(1)	SVAR	GC-VAR	DBNs
Score matrix	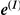	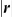	g	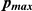
	Average time (second)			
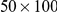	3.275	0.545	144.247	256.684
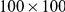	12.887	1.856	N/A	2065.752
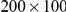	59.164	8.112	N/A	N/A
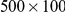	626.075	81.646	N/A	N/A

For the inferred network probability matrices, we compute their true positives (TP), false positives (FP), true negatives (TN), and false negatives (FN) at a given threshold. This procedure was repeated 500 times for each test statistic and variance scenario to obtain Receiver Operator Characteristic (ROC) curves [15], [16] for describing the dependence of true positive rate 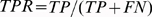 and false positive rate 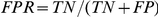. ROC curves provide a straightforward graphical representation of the performance of the algorithms. They are especially useful in comparisons by using many thresholds. As a summary metric for ROC, the area under the ROC curve (AUC), as its name indicates, measures the average accuracy of the prediction.

While AUC provides a quantitative measurement on the average performance for a method, maximum F-score [17] evaluates each method at its point of optimum performance. F-score is the harmonic mean of precision (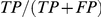) and recall (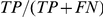). In the implementation we use a balanced harmonic mean of precision and recall. As a composite measure, the F-score penalises algorithms with higher specificity and rewards algorithms with higher sensitivity.

Apart from these metrics, we also base our evaluation on the consumed computation time and the true positive rate at a 0.2 false positive rate, since usually a low false positive rate is preferred. All three metrics are used for assessment in the simulation experiments.

#### Experimental results

For DPC and SVAR, we plot their experimental results together so that they can be compared with respect to individual simulations. Then the average results on part of the datasets for each of the four algorithms are shown in separate plots. This is because GC-VAR can only be applied on datasets with 100 time points and 50 genes, since it requires long time series (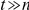) to fit linear models. Because of the high computational costs of DBNs, we only compute its results for networks of size 50 and 100 for both the 21 time point and 100 time points datasets.

**Figure 5:**
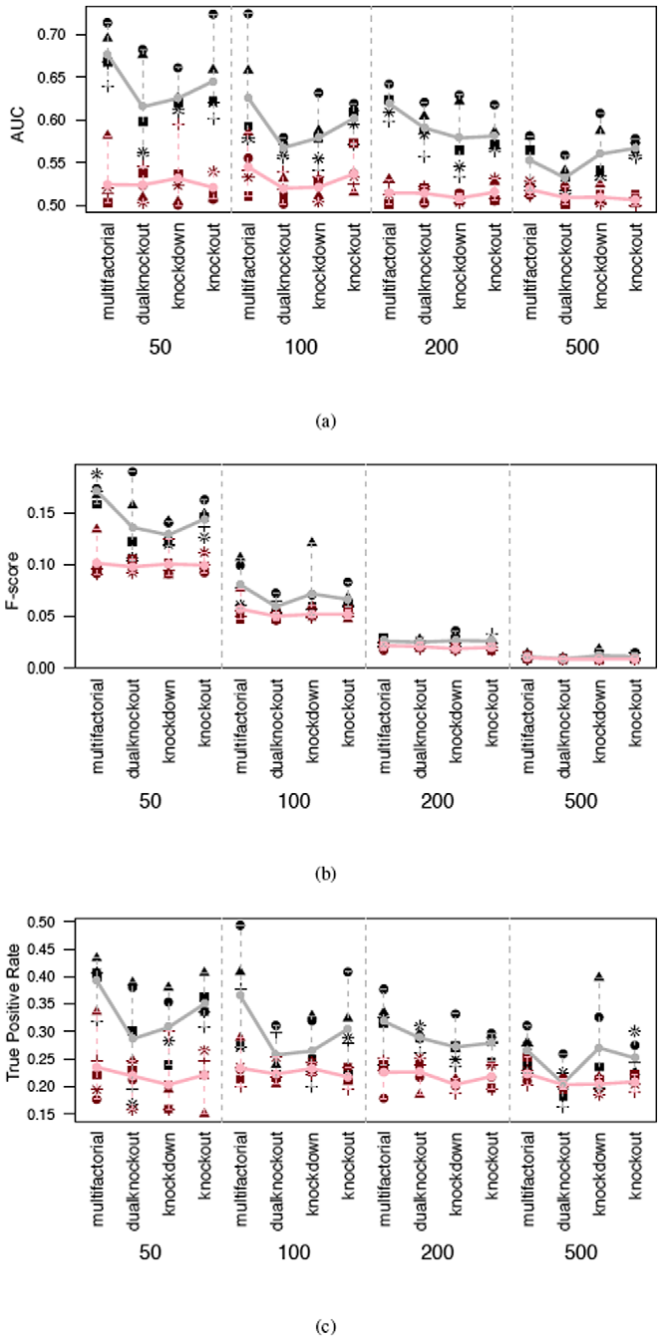
Performance scores of two network inference algorithms (black/grey: DPC, red/pink: SVAR) when tested on datasets with 50, 100, 200, and 500 genes respectively and 21 time points. The symbol denotes the identity of the five simulations, and the lines denote the average of simulation results. (a) AUC values, (b) F-score, (c) TPR at FPR of 0.2.

**Figure 6:**
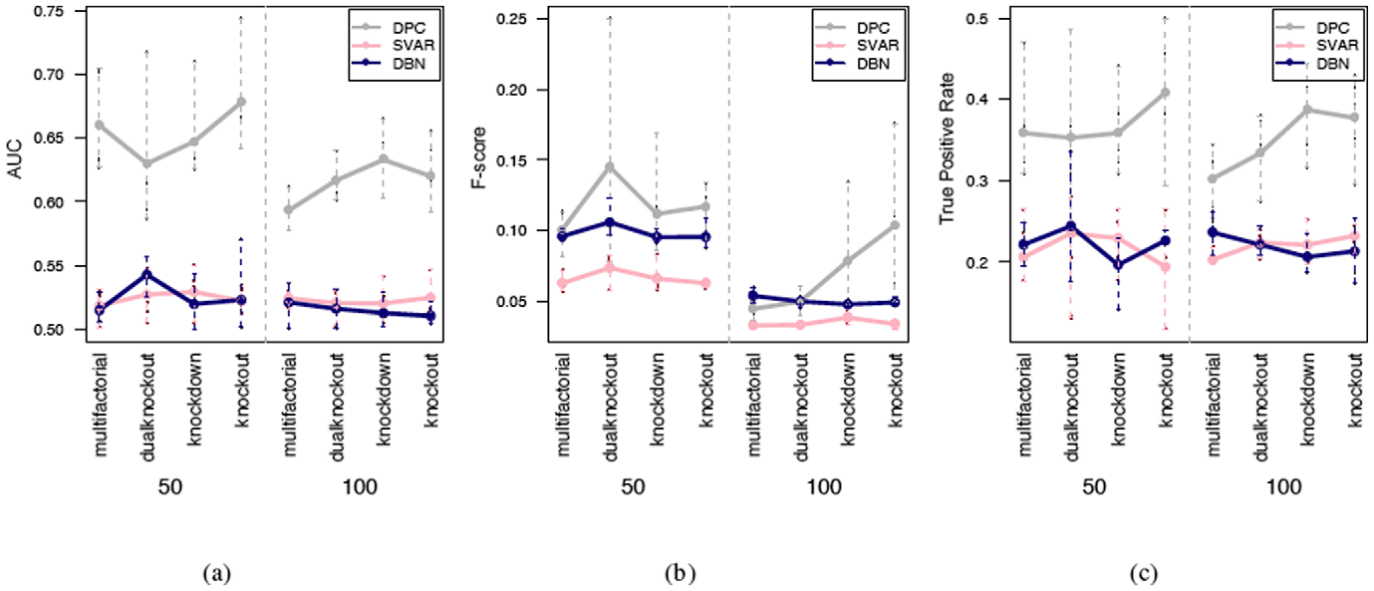
Performance scores of three network inference algorithms when tested on 50 and 100 gene networks with 21 time points. (a) AUC values, (b) F-score, (c) TPR at FPR of 0.2.

With 21 time points, quantitative measurements of performance including AUC values, and F scores and true positive rates at 0.2 false positive rate for SVAR and DPC are provided in Figure 5. It is easy to observe a descent in their performances as network size increases. This is expected for difficult inference tasks with low signal-to-noise ratio. In these simulations, the true signal as a result of the initialization of perturbation, often on a single gene, is easily buried among the experimental noise. Nevertheless, in comparison DPC shows superior performance in the results. Following, the results as the average of the outcome of simulations of same setting Figure 6 compares the performance of DPC, SVAR and DBN, with error bars showing the range of results. Here again, DPC achieves the best results.

**Figure 7:**
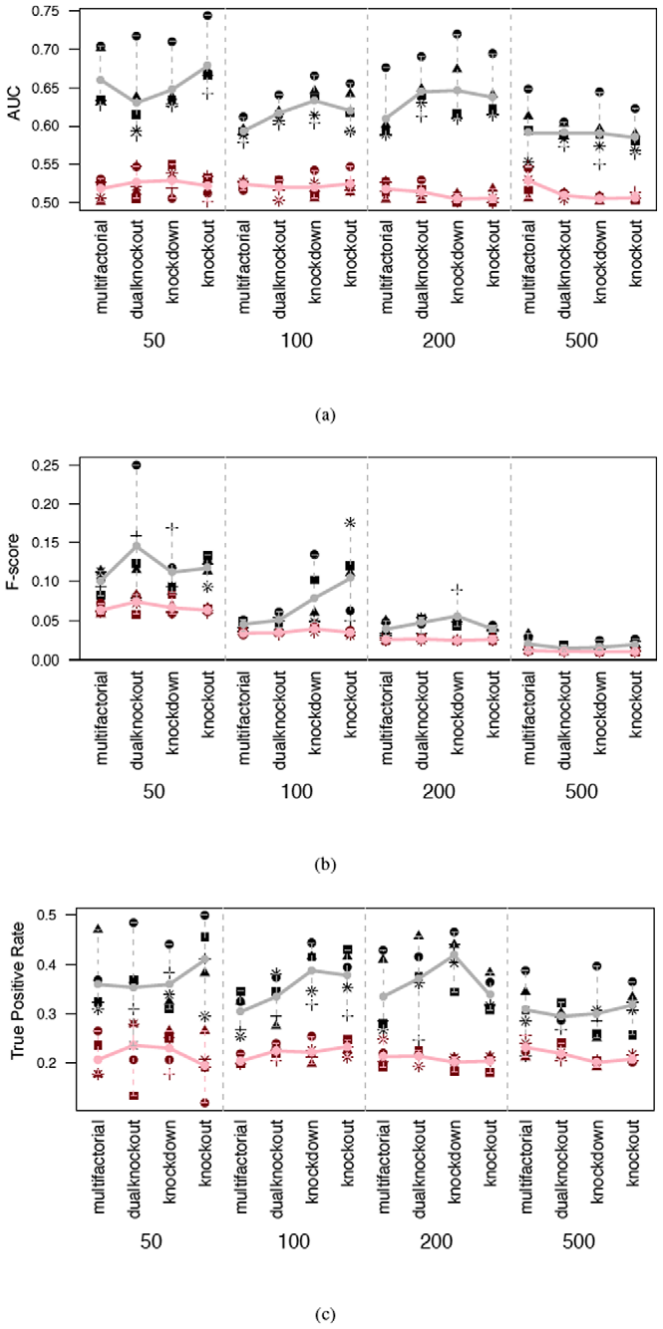
Performance scores of two network inference algorithms (black/grey: DPC, red/pink: SVAR) when tested on networks with 50, 100, 200, and 500 genes respectively and 100 time points. (a) AUC values, (b) F-score, (c) TPR at FPR of 0.2.

**Figure 8:**
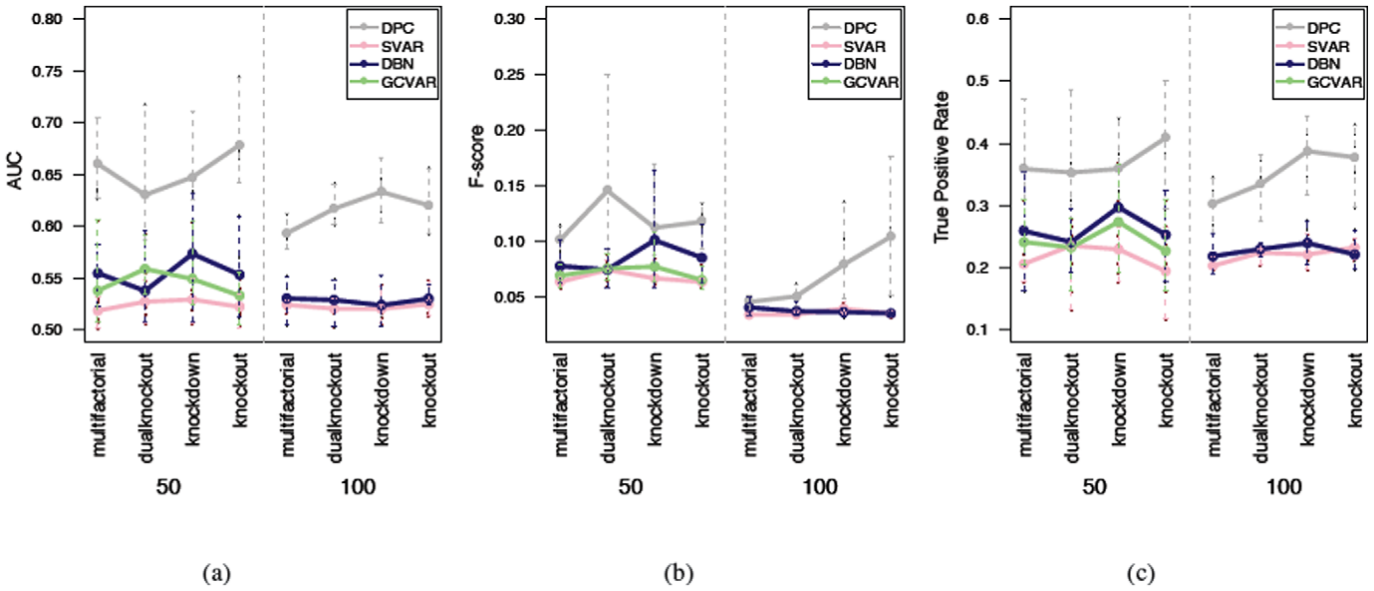
Performance scores of four network inference algorithms when tested on datasets of tested on 50 and 100 gene networks with 100 time points. (a) AUC values, (b) F-score, (c) TPR at FPR of 0.2.

With 100 time points, DPC also outperforms SVAR in terms of AUC, F-scores, and the true positive rates at 0.2 false positive rate (Figure 7). Comparing results from the 21 time point experiments (Figure 5) and the results from the 100 time points experiments (Figure 7), the influence of sample sizes on the performance can be observed. This conforms to current theory and is reassuring. Then measurements of the performance of all four methods on part of the 100 time point datasets is shown in Figure 8. From the average result, GC-VAR and DBN only outperform SVAR for the 50 gene network, while DPC is the best performer. The average consumed time for the 100 time point datasets is given in Table 1 on a Mac Pro (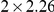 GHz). For the 21 time points datasets consumed time is similar with the results in this table and hence is not shown. From this table, a noticeable advantage of DPC can be seen in its efficiency, although SVAR is the most efficient.

#### A note on the performance

Several state-of-the-art methods have also been tested but showed poor performance on the GNW benchmarks (data not shown). This may be because some perturbations have only few downstream effects. When the regulators are not perturbed, the real relationships between them and their downstream targets cannot be found. Effectively, a significant proportion of the variation in the data is a result of experimental noise. Hence for some inference methods, it is difficult pick up the true signals amidst noise.

In DREAM an *in silico* challenge provides four perturbations each of 10 simulations to infer a network. However, for benchmarking in this paper we use a single simulation for one type of perturbation as input. As a result, better performance in DREAM can be expected for the methods in comparison. Nevertheless, for comparative purposes, these simulation assessments undoubtedly yield benchmarking results on a fair ground, i.e. not biased towards any model assumptions.

### Experiments on a biological dataset

To test these methods' performance on biological data, a *Arabidopsis L. Heynth* dataset [18] of 800 genes and 22 time points is used. The data is collected from an experiment investigating the impact of the diurnal cycle of the starch metabolism in the leaves of *Arabidopsis*. Two replicates consist of measurements at 11 time points of uneven time intervals to capture the periods immediately after the transitions from dark (light) to light (dark). Samples were firstly taken at the end of light period, then at 1, 2, 4, 8, and 12 hr of darkness and at 1, 2, 4, 8, and 12 hr of light. During the day, starch is synthesised to serve as an intermediate store of carbon fixed during photosynthesis when rates of production exceed the export rates of the chloroplasts. During the night, starch formed and stored within the chloroplasts during the day is metabolized to maltose and glucose and exported from the chloroplast. These exported breakdown products are then used as sources of energy for plant growth and metabolism as well as being sent to sink tissues where starch can be re-synthesised for more long term storage in specialised storage organelles called Amyloplasts (for a detailed review see [19]).

For the assessment of our validation scheme, a subset of 800 genes is used which was previously selected using a periodicity test [20] and was first studied by Rhein and Strimmer for gene network inference [1].

Given the sample size and network size, GC-VAR and DBN cannot be applied to this dataset. DPC and SVAR are applied to produce resulting probability matrices. We compare the probability matrices by using two validation methods as below. As the length of time series is short, we choose 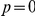 for DPC.

#### Validation with SAMBA for extracting network modules

**Figure 9:**
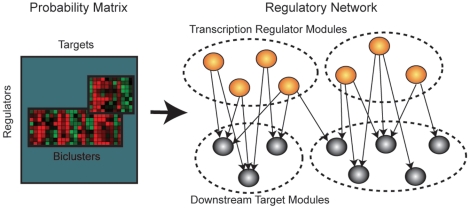
Biclustering a network probability matrix is equivalent to pulling out network modules. Because coefficients in the biclusters are highly correlated, it means genes in rows and columns share similar regulatory patterns.

A biclustering method is adopted as part of the validation process. Biclustering aims to find a group of variables that share similar data patterns under a subset of conditions. When applied to expression data, it searches for a group of genes with similar expression patterns under a subset of conditions/treatments. But when applied to probability matrices that indicate regulatory interactions, as described previously in [21], it searches for a subset of genes with similar regulatory patterns whilst under the regulatory influence of a second subset of genes, as illustrated in Figure 9. In other words, biclustering can be applied to probability matrices to get statistically significant sub-matrices, which are equivalent to network modules in our case.

With probabilistic modeling and graph theory techniques, SAMBA [22] identifies subsets of rows of a matrix that jointly respond in a similar manner across subsets of columns. The biclusters are allowed to be overlapped. In our experimental design, each value in the probability matrices indicates whether a gene corresponding to a row regulates a gene in a column. The resulting biclusters of the probability matrix correspond to regulatory network modules, with rows corresponding to groups of regulatory genes regulating sets of target genes in the columns. Therefore SAMBA provides an efficient way of validating network inference algorithms. One main advantage of this method is that, by allowing overlapping modules/biclusters, regulators and targets are allowed to appear in different network modules. This satisfies the biological assumption that genes may have multiple functions and can be involved in different pathways. Also, it allows multiple regulators to exert their potential regulatory influences in hierarchical or co-dependant manners on particular subset of targets.

247 biclusters were found for the DPC matrix with quality scores of 88–2277 (mean = 260.8, sd = 219.7), while 257 biclusters were found for SVAR with quality scores of 87–940 (mean = 233.5, sd = 102.8). GO [23] and promoter enrichment were computed for each of the biclusters, as listed in Table S1 and S2 for DPC and SVAR. In summary, the DPC biclusters are enriched with 47 GO terms, while the SVAR biclusters are enriched with 24 GO terms (corrected 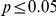). The fact that DPC presents more GO terms for its biclusters than SVAR suggests that DPC is inferring more fundamentally accurate regulatory interactions, which in turn results in biclusters/regulatory modules of targets which are more likely to be co-regulated members of the same biological process or pathway. Several GOs (chloroplast - GO:0009507; plastid - GO:0009536; organelle envelope - GO:0031967; organelle membrane - GO:0031090; organelle subcompartment - GO:0031984; and photosynthesis - GO:0015979) identified by both DPC and SVAR suggest that the biclusters represent modules of genes with potential roles in diurnal starch metabolism. In addition, one GO term identified in two biclusters from the DPC results and not SVAR is starch metabolic process - GO:0005982. This is a highly informative GO considering the biological process under investigation [18], suggesting that perhaps DPC is uncovering more biologically relevant gene-gene associations from the data.

Enrichment analysis is performed for transcription factor binding elements in target promotors. 60 cases of promotor enrichment were observed for DPC biclusters while 44 cases were identified in the SVAR biclusters (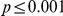). Lists of promotor enrichments are provided in Table S3 and S4 for DPC and SVAR. Again, in spite of the observation that SVAR produced more biclusters than DPC, the results suggest that DPC gives rise to more intuitive groupings of genes, as we would expect co-regulated genes to share common binding motifs in their promotors, where transcription factors that are involved in their co-ordinated regulation can bind.

#### An interesting starch deregulation bicluster

**Figure 10:**
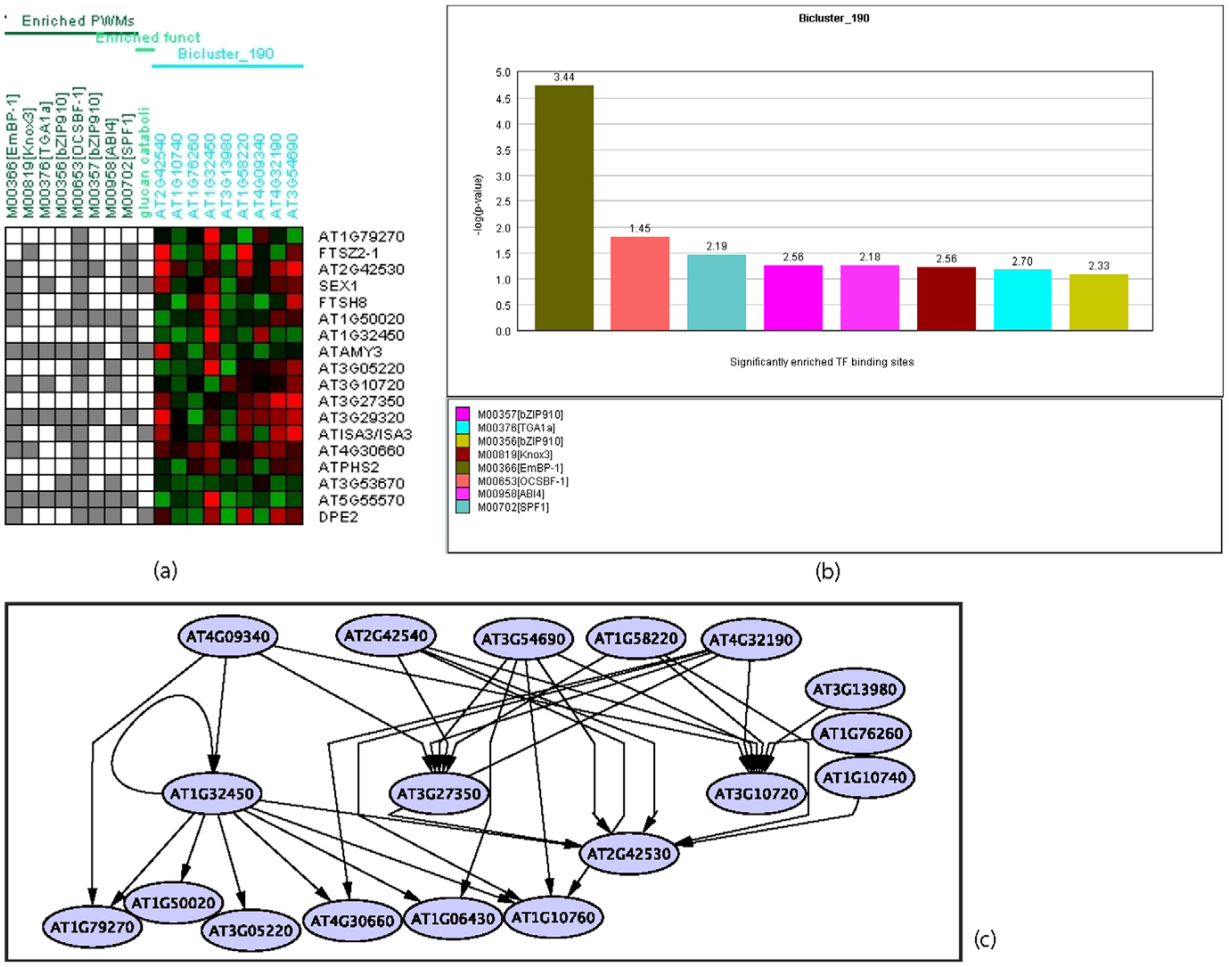
New information can be derived from a bicluster 190 combining biological knowledge and DPC network. (a) Heatmap of the bicluster number 190 of the DPC network probability matrix. (b) Significantly enriched motifs in this bicluster. (c) A visual representation of the interactions predicted by DPC(1) between the members of bicluster 190. While statistically not the strongest interactions resolved within the 800 gene set, the biological association of the co-regulated targets none the less indicates that the biclustering has helped to reveal weaker but far more pertinent signals of potential co-ordinated regulation. These relationships would otherwise have been lost if a rudimentary ranking of the strengths of the interactions had been used instead.

More interestingly, one bicluster of the DPC network was found to have several members of the starch degradation pathway active in the chloroplasts in the dark. This bicluster (number 190 as shown in Figure 10), has about 27 members where 5 of these (Figure 10(a)) are known to be involved in starch degradation and two more with potential involvement in this process due to familial relationships with known components of this pathway [19]. Of the 48 genes investigated in [18], 10 of these were included in this 800-gene dataset.

In particular, AMY3 (At1g69830), ISA3 (At4g09020), PHS1 (At 4g29320) and SEX1/GWD1 (At1g10760) are known to be part of the starch degradation pathway operating in chloroplasts [19]. In addition, two other members PHS2 (At3g46970) and DPE2 (AT2g40840) with their paralogous relationships to the pathway described above also present interesting information regarding their potential co-ordinated regulation with the four chloroplast components (Figure 10(c)). Furthermore, this cluster also contains COR15B (At2g42530) while its homologue COR15A (At2g42540) is a suggested regulator of the bi-cluster. Both proteins are induced by cold stress and abscisic acid treatment [24], while COR15A has been shown to be present in chloroplasts [25]. Another proposed regulator of this bi-cluster is the uncharacterised MYB transcription factor (At1g58220) which is interesting considering several MYBs have been implicated in regulating photosynthesis under stress [26].

The tight grouping of these genes within the large background subset of genes indicates that DPC appears to be identifying target genes which are potentially co-regulated and involved in the same biological pathway. A larger bicluster (number 222), containing 43 members identified within the SVAR data was also found to contain 3 of the 5 known genes and the two putative pathway members identified in the DPC bicluster number 190. From the validation results it appears that DPC generates more biologically meaningful results than SVAR.

One of the significantly enriched motifs in bicluster 190 one motif M00958 (Figure 10(b)) suggests that ABI4, a transcription factor known to influence photosynthesis and starch regulation in response to ABA and sugar signalling [27] may play and important regulatory role. There is also a further interesting level of correlation given that the proposed target COR15B and the proposed regulator COR15A in this bicluster are both transcriptionally induced following exogenous application of ABA [24].

#### Validation with transcription regulators as network hubs

Besides analysing the biclustering results, we also looked directly for known regulators. 41 of the 800 genes are known transcription factors in Arabidopsis. Since a row of coefficients in the probability matrix represents probabilities of one gene regulating other genes, the sum of this row should be proportional to the probability of this gene being a regulator. A Welch-Satterthwaite test [28] is performed to compare two groups with the alternative hypothesis that the mean of one group is greater than the other: the rows of the probability matrix for the 41 transcription factors is compared against all of the probability matrix. The more significantly different the two groups are, the better the probability matrix differentiates between known transcription factors and other genes. Therefore, the method that better captures the underlying network structure is the one with the more significant result from this t-test. The resulting p-values are 1.0e-14 for SVAR and 2.2e-16 for DPC, indicating that DPC is better in capturing the network structure with respect to these hubs.

The 41 transcription factors are then tested individually for their roles in the probability matrices of DPC and SVAR in the same way. Results of the test are provided in Table S5 and S6 for DPC and SVAR, respectively. The well known circadian regulators LHY and CCA1 [29], [30] are regarded as transcription factors by DPC (both have corresponding 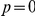). Test results for SVAR are 0.358 and 0.927, respectively. Further, promoter analysis in the region of −1,000 to 200 bp of the downstream targets is performed for both of the potential hubs. To conduct the comparison on a fair ground, we take the 30 most significantly interacting genes with LHY and CCA1 in both cases for SVAR and DPC. For SVAR, promoters of putative CCA1 targets are enriched with 7 motifs with p-values ranging from 1.2E-14 to 9.3E-12. Promoters of putative LHY targets are enriched with 10 motifs with p-values from 1.3E-14 to 7.6E-12. For DPC, promoters of putative CCA1 targets are enriched with 6 motifs with p-values ranging from 1.4E-14 to 7.5E-12, and promoters of putative LHY targets are enriched with 10 motifs with p-values from 1.0E-15 to 4.7E-12. In particular, the motif with p-value 1.0E-15 in promoters of putative LHY targets (Figure S1), as determined by DPC, is close to a known motif HSF(M00028). As both of these methods identified similar numbers of motifs no real conclusion can be drawn as to which method is superior in this respect, as both methods may have uncovered equally valid binding motifs. Here we are also limited by the number of biologically determined motifs for which there exist probability weight matrices. Such that the presence of the known motif HSF(M00028) should not mitigate the importance of the other motifs which are equally plausible until otherwise experimentally disproved. Nonetheless the above analyses of the biological dataset have presented many interesting possibilities concerning the transcriptional regulation of diurnal starch metabolism which warrant further experimental investigation. Overall, DPC appears to reveal more biologically intuitive and plausible regulatory scenarios.

## Discussion

This paper reviews some recent advances in multivariate time series inference of gene expression data. It then reports a new method, Directed Partial Correlation (DPC), for efficient and effective large-scale network inference. Experiments on both simulated and biological data are designed to investigate the properties of the proposed method and existing methods.

From the experimental results, superior performance of the proposed DPC method is observed when compared to three other inference methods. When analyzing simulated datasets, DPC can pick up the true signal and reveal the underlying relationships. SVAR is the most efficient, but less effective than DPC in most of the cases. For the biological dataset, DPC appears to give more biological meaningful results than SVAR. These results provide good evidence that DPC is suitable for the scenario of expression time series analysis.

Additionally, we should be aware that high-throughput data often lacks the specificity for accurate inference of regulatory relationships. Therefore, the network inference result can be either examined in a modular fashion as in the paper, or combined with other data sources or biological knowledge to address complex biological problems.

In summary, the proposed DPC algorithm has excellent performance with large numbers of variables. Its efficiency in learning among hundreds of variables is mainly due to the fact that the computation is based on partial correlation instead of model fitting. DPC has the potential of being extended to applications on static data such as cancer expression for learning the data structure. With time series data, the time lag should be carefully selected based on users understanding of the dataset, in order to reveal the information embedded in time lags.

## Supporting Information

Figure S1 A significantly enriched motif in LHY targets as determined by DPC in network module/bicluster 190.
(TIF)

Table S1GO enrichment for DPC biclusters (Bonferroni adjusted 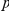 -value 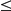 0.05).(XLS)

Table S2GO enrichment for SVAR biclusters (Bonferroni adjusted 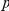 -value 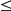 0.05).(XLS)

Table S3Transcription factor enrichment for DPC biclusters (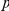 -value 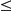 0.001).(XLS)

Table S4Transcription factor enrichment for SVAR biclusters (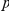 -value 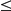 0.001).(XLS)

Table S5 Transcription regulators ranking by SVAR.
(TXT)

Table S6 Transcription regulator ranking by DPC(1).
(TXT)
