# Supplementary figures and images for: Directed Partial Correlation: Inferring Large-Scale Gene Regulatory Network through Induced Topology Disruptions
Source: PLoS One. 2011 Apr 6;6(4):e16835. doi: 10.1371/journal.pone.0016835 (PMC3071805; doi:10.1371/journal.pone.0016835)

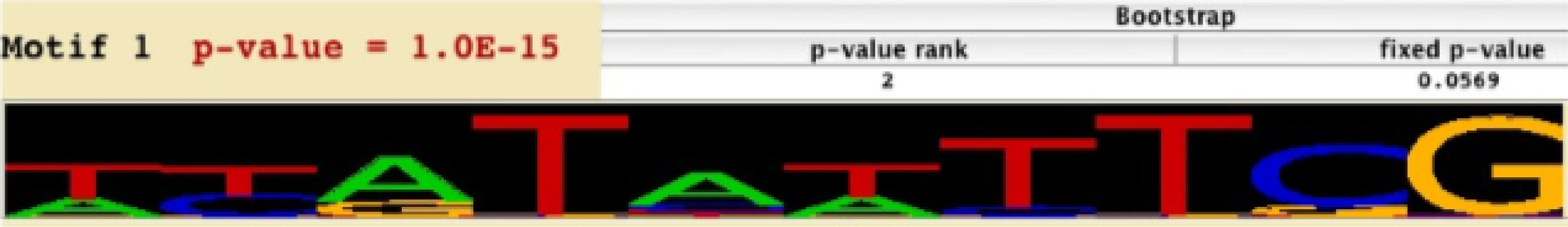

Supplement: Figure S1 — A significantly enriched motif in LHY targets as determined by DPC in network module/bicluster 190. (TIF) [file pone.0016835.s001.tif]
